# The Role of Preoperative Educational Intervention in Reducing Parental Anxiety

**DOI:** 10.7759/cureus.26548

**Published:** 2022-07-04

**Authors:** Rafia Afzal, Saima Rashid, Fauzia A Khan

**Affiliations:** 1 Anaesthesiology, Aga Khan University Hospital, Karachi, PAK

**Keywords:** paediatric patients, patient education, preoperative care, visual analogue scale, anxiety

## Abstract

Background and objective

The parents of pediatric patients admitted for elective surgery exhibit significant levels of anxiety. The reduction in parental anxiety is directly proportional to the information and counseling provided to the parents preoperatively. The parenting style in South Asian culture is different from that of western cultures and may influence the response to these interventions. In this study, we aimed to compare the mean anxiety levels between parents of children (aged three to eight years) undergoing outpatient infraumbilical surgery equipped with standardized preoperative parental education and those without.

Methods

This was a randomized, controlled, and blinded trial. Parents of 72 pediatric patients (aged three to eight years) undergoing elective infraumbilical daycare surgery were enrolled and were divided into two groups: an intervention (Group I) and a non-intervention (Group NI) group. Both groups received routine verbal counseling at the preoperative clinic, but a standardized brochure was provided only to Group I. Parental anxiety was measured by using the Visual Analog Scale (VAS) at three different time points: in the outpatient surgery suite on the day of surgery, in the preoperative area 10 minutes before shifting the child to the operating room, and finally in the recovery room.

Results

The baseline mean VAS score was significantly higher in Group I compared to Group NI (p=0.017). After the intervention, the mean pain score significantly decreased from baseline in Group I as compared to Group NI (mean ±SD: 4.08 ±1.6 vs. 6.08 ±1.66; p=0.0005).

Conclusion

The information provided through standardized written material to the parents on the day of surgery before anesthesia helped to significantly attenuate preoperative parental anxiety.

## Introduction

Parental anxiety is a common response encountered during the preoperative assessment of pediatric patients. Around 47% of the parents of pediatric patients admitted for elective surgery exhibit anxiety-related illness [1]. Excessive parental apprehension preoperatively can lead to increased anxiety in children, which can result in negative postoperative behavior [2]. Reduction in parental anxiety is directly proportional to the information and counseling provided to the parents preoperatively [3].

Ethnicity, language, culture, and socioeconomic status can all influence parental behavior [4]. In this study, our aim was to assess the impact of preoperative education on reducing parental anxiety among Pakistani parents whose children were scheduled for surgery. There is scarce data on this topic in the published literature from our part of the world. We used the Visual Analogue Scale (VAS), a validated tool, to measure preoperative anxiety [5,6].

The objective of this study was to compare the mean anxiety levels of parents of children undergoing infraumbilical daycare surgery who were provided a printed information brochure in the preoperative clinic versus those of a control group at three different time points. Both set of parents were provided conventional verbal information by the attending anesthesiologists in the preoperative clinic.

## Materials and methods

This randomized controlled trial was conducted between January 1 and March 31, 2019, at the Aga Khan University Hospital after obtaining approval from the Institutional Ethical Review Committee (ERC) (5107-Ane-ERC-17). Written informed consent was taken from the parents of the participants. The primary endpoint of this study was preoperative parental anxiety as assessed by the VAS. The sample size calculation was based on a previous study [7] that used the VAS score (mean ±SD: 42.8 ±14) of parents in the control group. A 25% difference in the anxiety levels between the intervention and control groups was considered clinically significant. A sample size of 29 subjects in each group was deemed sufficient to detect a 25% difference in anxiety levels with a power of 90% and 5% alpha level. We recruited 72 subjects (36 in each group) to adjust for an assumed 20% dropout rate.

All parents of children of either gender aged between three to eight years with normal developmental milestones, with a minimum educational level of grade 10 (matriculation) were included. All children were scheduled to undergo infraumbilical surgeries in the outpatient surgery suite. Parents of children with chronic illnesses and redo surgeries were excluded from the study.

The recruitment was done in the preoperative clinic by a person blinded to the study. The randomization was performed by using sequentially numbered, opaque sealed envelopes (SNOSE) to assign subjects to one of the following two groups; intervention (Group I) or non-intervention (Group NI) group. Conventional verbal information regarding anesthesia was provided to both groups of parents in the preoperative clinic by the anesthesia team, while the written information brochure was only given to the parents in Group I (on the day of surgery in the daycare suite). The brochure comprised written information regarding the preoperative area, induction of anesthesia, emergence, and the post-anesthesia care unit (PACU). The criteria for discharge from PACU were also addressed. Parents were instructed on filling out the forms and VAS scores by the principal investigator.

Parental anxiety was measured using VAS. Additional details that were documented included the demographic data and the socioeconomic information of the parents, the American Society of Anesthesiologists (ASA) physical status classification, and the planned surgical procedure. The distribution of written information brochures and distribution/collection of VAS forms from the parents were done by two different individuals so that the blinding of outcome assessment was ensured.

The VAS scores were recorded at three different time points. The first time was in the daycare suite on the day of surgery, after parents were allocated to either Group I or NI. This value was taken as the baseline. Then, the parents in Group I were given the educational brochure to read over.

Parental anxiety was measured for the second time when the parents were present in the preoperative area with their children; this was around 10 minutes before the child was taken into the operating room. The third measurement was done in the PACU after the child was shifted there from the operating room. Three different forms were used to measure parental anxiety in three different areas to prevent the previous scores from influencing the current score.

Statistical analysis

Data were analyzed by using SPSS Statistics version 19 (IBM, Armonk, NY). Categorical variables such as gender, ASA status, and parents’ demographic data (i.e., gender, qualification, and occupation) were reported as frequencies and percentages. VAS scores were estimated in terms of mean and standard deviation with a 95% confidence interval for both groups. The independent sample t-test was applied to compare the mean VAS score between the groups. A p-value lower than 0.05 was considered statistically significant. Repeated measure analysis of variances (ANOVA) was also applied to compare within and between the subjected effects. Parental gender, qualification, and occupation were treated as confounders, and stratification analysis was performed to observe the effect of confounders on the anxiety score. The unpaired t-test was applied to observe the significant effect with the above-defined significant criteria.

## Results

During the study period, a total of 72 children were recruited and completed the study. The demographic data of the patients and details of parental education and occupation are listed in Table 1. Details regarding parental presence at induction and data on the surgical procedures are presented in Table 2.

**Table 1:** Demographic variables of children and educational level of parents I: intervention; NI: non-intervention; SD: standard deviation; ASA: American Society of Anesthesiologists

Variables	Group I (n=36)	Group NI (n=36)	P-value
Age (years)	4.36 ±1.47	4.54 ±1.79	0.64
mean ±SD			
Gender, n (%)			
Males	29 (80.6%)	30 (83.3%)	0.75
Females	7 (19.4%)	6 (16.7%)	
ASA classification			
I	27 (75%)	31 (86.1%)	0.23
II	9 (25%)	5 (13.9%)	
Educational level of parents, n (%)			
Matriculation (grade 10)	4 (11.1%)	8 (22.2%)	0.53
Intermediate (grade 12)	10 (27.8%)	9 (25%)	
Graduation	15 (41.7%)	15 (41.7%)	
Post-graduation	7 (19.4%)	4 (11.1%)	

**Table 2:** Parental presence at induction and surgical procedures performed I: intervention; NI: non-intervention

Variables	Group I (n=36), n (%)	Group NI (n=36), n (%)	P-value
Parental presence			
Father	17 (47.2%)	20 (55.6%)	0.479
Mother	19 (52.8%)	16 (44.4%)	
Procedure			
Inguinal hernia repair	9 (25%)	11 (30.6%)	0.599
Circumcision	12 (33.3%)	11 (30.6%)	0.800
Cystoscopy	5 (13.9%)	3 (8.3%)	0.453
Hypospadias repair	3 (8.3%)	3 (8.3%)	0.999
Orchidopexy	3 (8.3%)	2 (5.6%)	0.643
Orchidectomy	1 (2.8%)	4 (11.1%)	0.164
Sigmoidoscopy	2 (5.6%)	2 (5.6%)	0.999
Others	3 (8.3%)	7 (19.4%)	0.173

**Figure 1:**
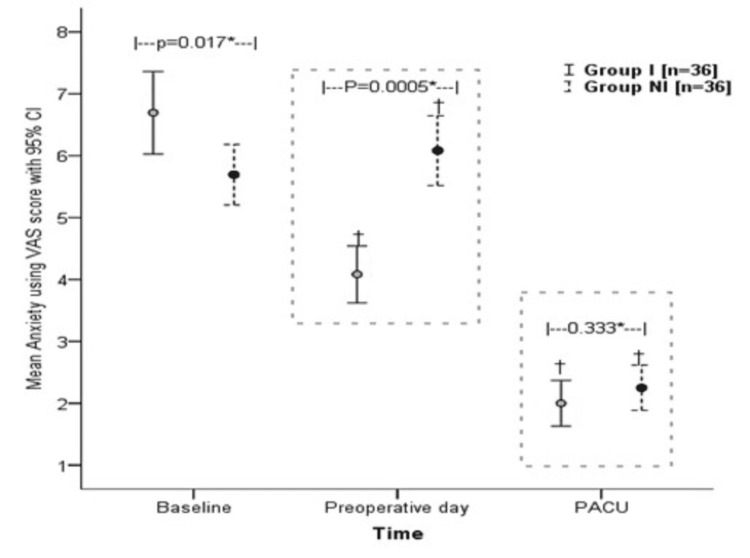
Comparison of mean anxiety scores between and within groups at three different time points I: intervention; NI: non-intervention; VAS: Visual Analog Scale; PACU: post-anesthesia care unit

The mean baseline anxiety score (VAS score) before the intervention was higher in Group I (mean ±SD: 6.69 ±1.97) compared to 5.69 ±1.45 in Group NI. This difference was statistically significant (p=0.017) despite randomization. However, the mean VAS score was significantly lower in Group I compared to Group NI after the intervention was done and the parents were analyzed in the preoperative bay: 4.08 ±1.36 in Group I vs. 6.08 ±1.66 in Group NI (p=0.0005). Furthermore, the mean VAS score of the parents in the PACU decreased from the baseline but did not show a statistically significant difference when the two groups were compared (mean ±SD: 2.00 ±1.09 vs. 2.25 ±1.08; p=0.333), as shown in Table 3 and Figure 1.

**Table 3:** Mean anxiety scores in both groups at three different time points I: intervention; NI: non-intervention; PACU: post-anesthesia care unit

Time of the estimation of the anxiety score	Groups	N	Mean	Standard deviation
Baseline anxiety score	Group I	36	6.69	1.969
	Group NI	36	5.69	1.451
Preop bay anxiety score	Group I	36	4.08	1.36
	Group NI	36	6.08	1.663
PACU anxiety score	Group I	36	2.00	1.095
	Group I	36	2.25	1.079

**Figure 2:**
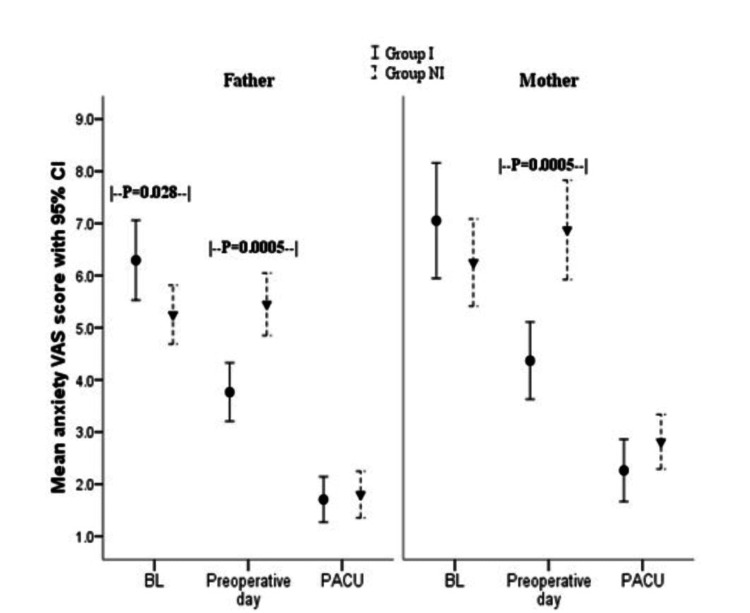
Comparison of anxiety scores between groups by parental gender I: intervention; NI: non-intervention; VAS: Visual Analog Scale; PACU: post-anesthesia care unit

The parental gender also influenced the mean preoperative anxiety score. The mean anxiety score was higher among mothers than fathers of the recruited subjects (mean ±SD: 4.91 ±1.04 vs. 4.05 ±1.52; p=0.004). But the mean anxiety score was significantly lower in the mothers in Group I when compared with Group NI in the preoperative area (p<0.05), as shown in Figure 2.

**Figure 3:**
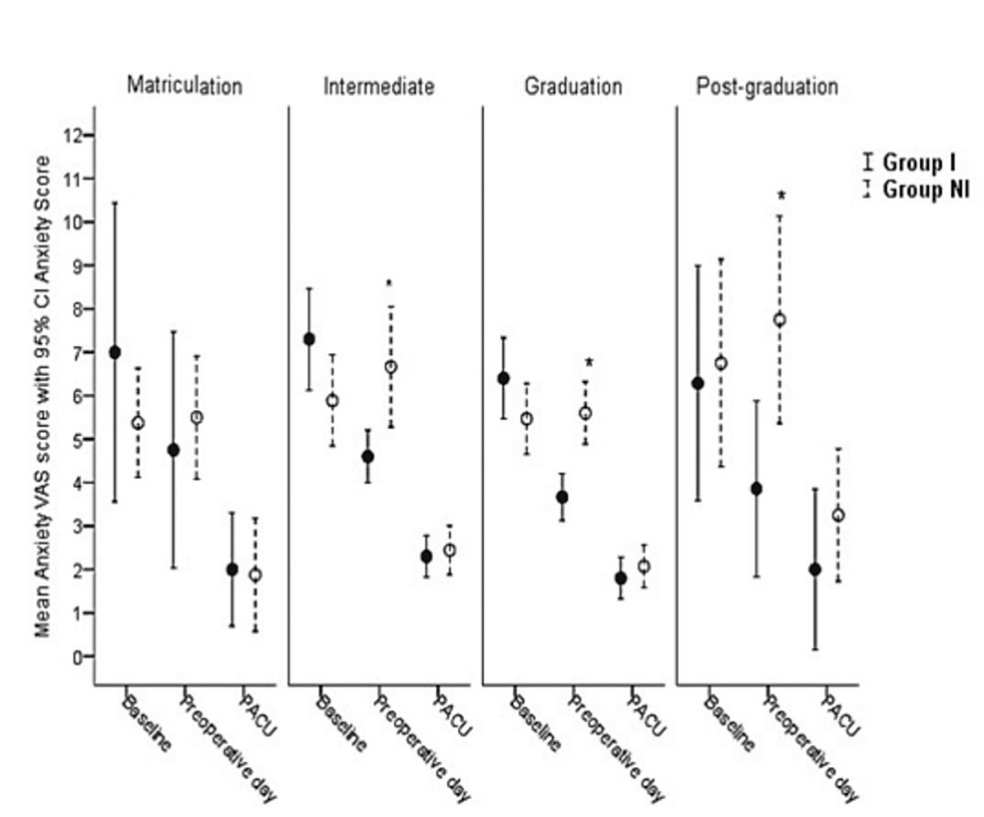
Comparison of anxiety scores between groups after controlling for the education level of parents I: intervention; NI: non-intervention; VAS: Visual Analog Scale; PACU: post-anesthesia care unit

No significant difference was observed in the baseline anxiety scores in terms of the level of education or qualification of the parents. Baseline anxiety scores of parents possessing low (till grade 10 or 12), average (graduation), and higher education (post-graduation) levels were as follows: grade 10: mean ±SD: 5.92 ±1.83; grade 12: 6.63 ±1.64; graduation: 5.93 ±1.62; and post-graduation: 6.45 ±2.42. However, there was a significant reduction in the mean anxiety scores noted in the preoperative area in Group I compared with Group NI after controlling for the effect of parental qualification (p<0.05), as shown in Figure 3.

## Discussion

We measured parental anxiety at three different time points in this study. Our study revealed that providing additional education to parents of children undergoing outpatient anesthesia with the help of a standardized brochure, earlier on the day of surgery, resulted in a 39% reduction in their anxiety before their children were shifted to the operating room [7,8,9]. Although similar studies are available on the subject in the literature, these have mostly been conducted in Europe and North America. The parenting style in South Asian cultures is different from these countries. Our hypothesis was those cultural variations and different parenting styles may give us different results since these could affect parental baseline anxiety and coping strategies differently. The response of the parents to different intervention tools may also be different, and hence it is important to observe the effect of interventions in different countries and different cultures.

Raghavan et al., in a study conducted in the US, compared the adaptive model of parenting in South Asian families living in Los Angeles with Euro-American families having developmentally delayed children. The two groups/ethnicities were found to be dissimilar in five domains and hence their coping mechanisms were also different. The domains included family support (which lacks greatly in the US), spousal relations (improving after childbirth in South Asian families), gender roles (mothers predominantly taking care of their children), cultural identity, and spirituality [10]. In our literature search, we could find only one similar study from a neighboring country, Iran, which was conducted by Tabrizi et.al., who found a 25% reduction in mean anxiety scores (VAS score) in the intervention group after an educational intervention was provided [11]. Our study showed a higher reduction in the mean anxiety of parents with a similar intervention.

We used the VAS, which is a validated tool, to measure parental anxiety. Various other methods have also been used to educate the parents preoperatively and ultimately to improve children’s preoperative anxiety. These include the use of videotapes, photo files, information brochures, pamphlets, demonstration of equipment using a role-modeling approach, and a PACU tour [12-15]. None of these intervention strategies has been proven to be superior to the others.

We also observed that the parental gender had an effect on the mean anxiety score. We saw higher mean anxiety scores in mothers of the recruited subjects. Pomicino et al. and Scrimin et al. have also observed that mothers were more anxious as compared to fathers about their child’s anesthesia and surgery [16-18]. Hence, focusing on the mothers and alleviating their anxiety may help in achieving positive outcomes [19].

In line with Shirley et al., we found that baseline anxiety was higher in housewives than in their working counterparts [20]; however, this anxiety was significantly reduced by the intervention. It was further observed that the level of education of mothers also influenced the anxiety scores. It will also be of value to observe the effect of different techniques for the reduction of preoperative anxiety and compare these with the educational level of parents and then see their impact on the behavior of the children.

This study has a few limitations. Primarily, we did not observe the effect of this reduction in parental anxiety on the children’s anxiety levels. We also did not standardize the gender of the parent accompanying the patients. Future research should try to identify the parental subgroups in which specific intervention strategies can be effectively applied. Also, there is a scope for repeating these studies in different cultures and subcultures to see if certain unique cultural characteristics influence parental anxiety levels.

## Conclusions

In this study, we aimed to compare the mean anxiety levels of parents of children undergoing infraumbilical daycare surgery (aged three to eight years) who were provided with a printed information brochure in the preoperative clinic (Group I) versus those of a control group who were not provided with the same (Group NI). The printed brochure comprised written information regarding the preoperative area, induction of anesthesia, emergence, and PACU. Based on our findings, the information provided through standardized written material to the parents on the day of surgery before anesthesia helped to significantly alleviate their preoperative anxiety.
